# 103. Quantifying the Time Required to Administer OPAT Care: A Missed Opportunity to Compensate for the Value of ID

**DOI:** 10.1093/ofid/ofad500.019

**Published:** 2023-11-27

**Authors:** Asher J Schranz, Michael Swartwood, Renae Boerneke, Teresa M Oosterwyk, Claire E Farel, Alan C Kinlaw

**Affiliations:** University of North Carolina, Chapel Hill, NC; University of North Carolina Medical Center, Chapel Hill, North Carolina; UNC Health, Chapel Hill, North Carolina; UNC Medical Center, Chapel Hill, North Carolina; UNC Chapel Hill, Chapel Hill, North Carolina; University of North Carolina School of Pharmacy, Chapel Hill, North Carolina

## Abstract

**Background:**

Outpatient parenteral antimicrobial therapy (OPAT) programs facilitate high-level care in the home and are linked to shorter hospitalizations and lower costs. Infectious diseases (ID) providers are concerned about lack of financial support and coordination for OPAT, which involves care and communication occurring outside of clinic visits. We described time devoted to OPAT care not directly remunerated via billable services.

**Methods:**

We included all courses in the UNC OPAT program, which mostly provides home-based OPAT, from 4/28/2020 (the start of the first course with available data on time) to 11/3/2022. OPAT providers logging time were a pharmacist, nurse coordinator, advanced practice providers (APP) and supervised trainees. Self-estimated time was documented by the provider at the time of service. “OPAT time” included lab monitoring, onboarding calls, and addressing symptoms or unanticipated events, but not physician or APP clinic visits, or time spent coordinating OPAT prior to discharge. OPAT time per OPAT course and clinical data were abstracted from medical records.

**Results:**

During the period, 969 courses had OPAT time recorded (approximately 40 concurrent OPAT courses at any given point in time). The median patient age was 57 and 58% were male. The most common diagnoses were osteomyelitis (40%), bacteremia (19%) and diabetic foot infection (12%), and the median OPAT course was 34 days (IQR 23-40). Patients averaged 1.5 clinic visits during an OPAT course. Median total hours of OPAT time per course was 2 (IQR 1.5-3, range 0.1-14.3) with numerous outliers accounting for substantial OPAT time (Fig 1-2). The median OPAT time per patient-week was 27 minutes (IQR 19-39, range 2-231), and ranged from 22 to 34 minutes in each quarter (Table). There were 2331 hours of OPAT time during the study period, an average of 18 hours per week.

**Figure 1:**
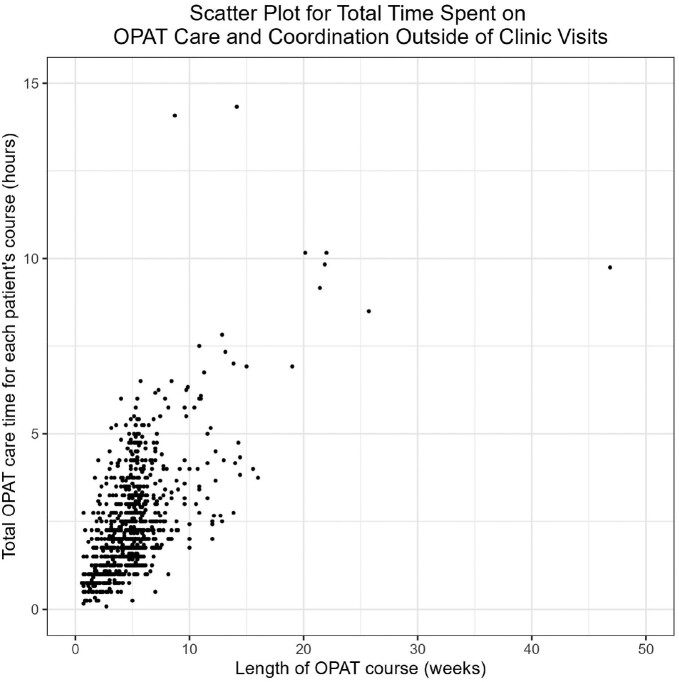
Total Time Spent on OPAT Care and Coordination Outside of Clinic Visits Scatterplot of total time spent on OPAT care and coordination outside of clinic visits ("OPAT time") per OPAT course, by length of OPAT course. All 969 OPAT courses are included.

**Figure 2:**
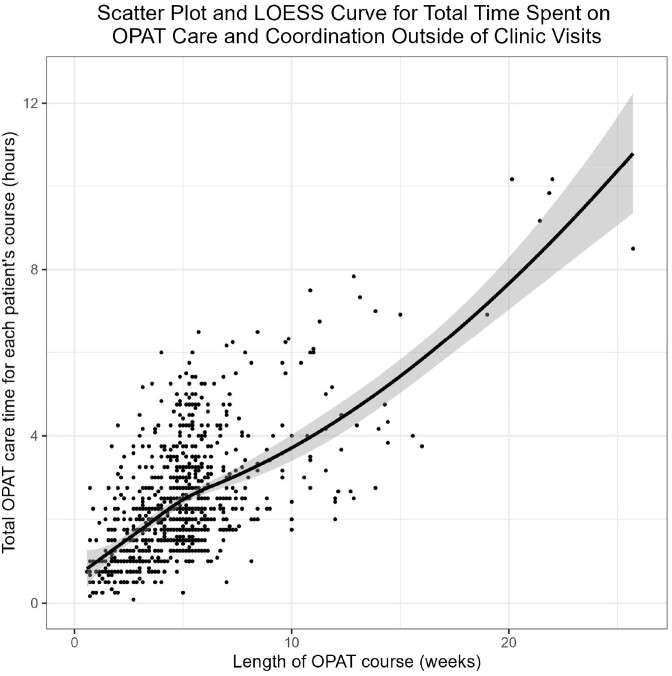
Trend of Time Spent on OPAT Care and Coordination Outside of Clinic Visits, with Outliers Removed LOESS curve trendline of total time spent on OPAT care and coordination outside of clinic visits ("OPAT time") per OPAT course, by length of OPAT course. The three most extreme outliers are removed.

**Table:**
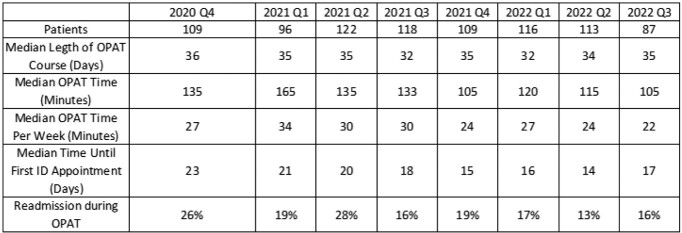
Quarterly Characteristics of Included OPAT Courses and Time Spent on OPAT Care

**Conclusion:**

OPAT services rely on substantial unreimbursed care time, consistent with weekly Level 3 established clinic visits for a median 5 weeks. Although a PharmD and RN largely provided services, many comparable programs are structured for physicians and APPs to provide the services of OPAT time. This data supports the development of alternative payment models, such as bundled payments, to compensate OPAT, an ID service that provides high-value care.

**Disclosures:**

**Asher J. Schranz, MD, MPH**, WoltersKluwer: Honoraria **Alan C. Kinlaw, PhD, MSPH**, Genentech: Grant/Research Support

